# Supplementary material for: Structural Characterization of Native RNA Polymerase II Transcription Complexes and Nucleosomes in Drosophila melanogaster
Source: Nat Commun. 2026 Aug 5;17:9616. doi: 10.1038/s41467-026-75963-0 (PMC13554098; doi:10.1038/s41467-026-75963-0)
Supplement: Supplementary file 1 [file 41467_2026_75963_MOESM1_ESM.pdf]

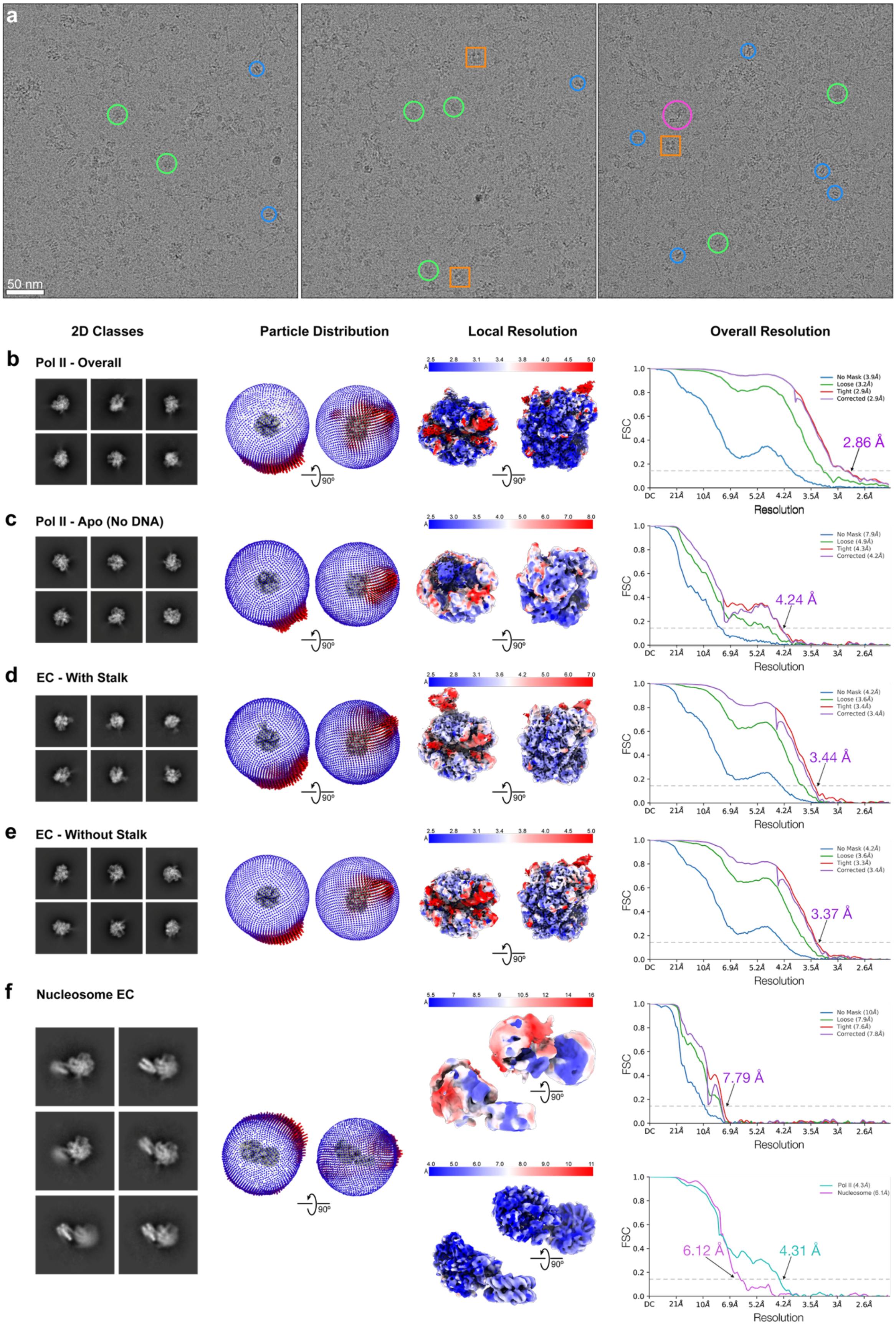
a
50 nm
2D Classes
Particle Distribution
Local Resolution
Overall Resolution
b
Pol II - Overall
2.86 Å
c
Pol II - Apo (No DNA)
4.24 Å
d
EC - With Stalk
3.44 Å
e
EC - Without Stalk
3.37 Å
f
Nucleosome EC
7.79 Å
6.12 Å
4.31 Å
FSC
Resolution

Supplementary Figure 1. Cryo-EM micrograph and analysis.

(a) Representative cryo-EM micrographs showing heterogeneity of the affinity-purified Pol II complex. Examples of different types of particles are marked: polymerases (green circle), nucleosomes (blue circles), nucleosome EC (pink circle), DLST (orange square) **(b-f)** Statistics on final reconstructions of apo-form Pol II (**b**) and ECs (**c-f**) including selected 2D class averages (left), Euler angle distribution of particles used in final reconstructions (second to left), local resolution maps with color ranging from blue to red representing high to low resolutions (second from right), and FSC curves calculated in CryoSPARC reported at the 0.143 FSC cutoff (right). In the Euler angle plot, the length of the cylinders is correlated to the distribution of particles in that direction. In the local resolution visualization, the maps are colored locally according to the scale above.

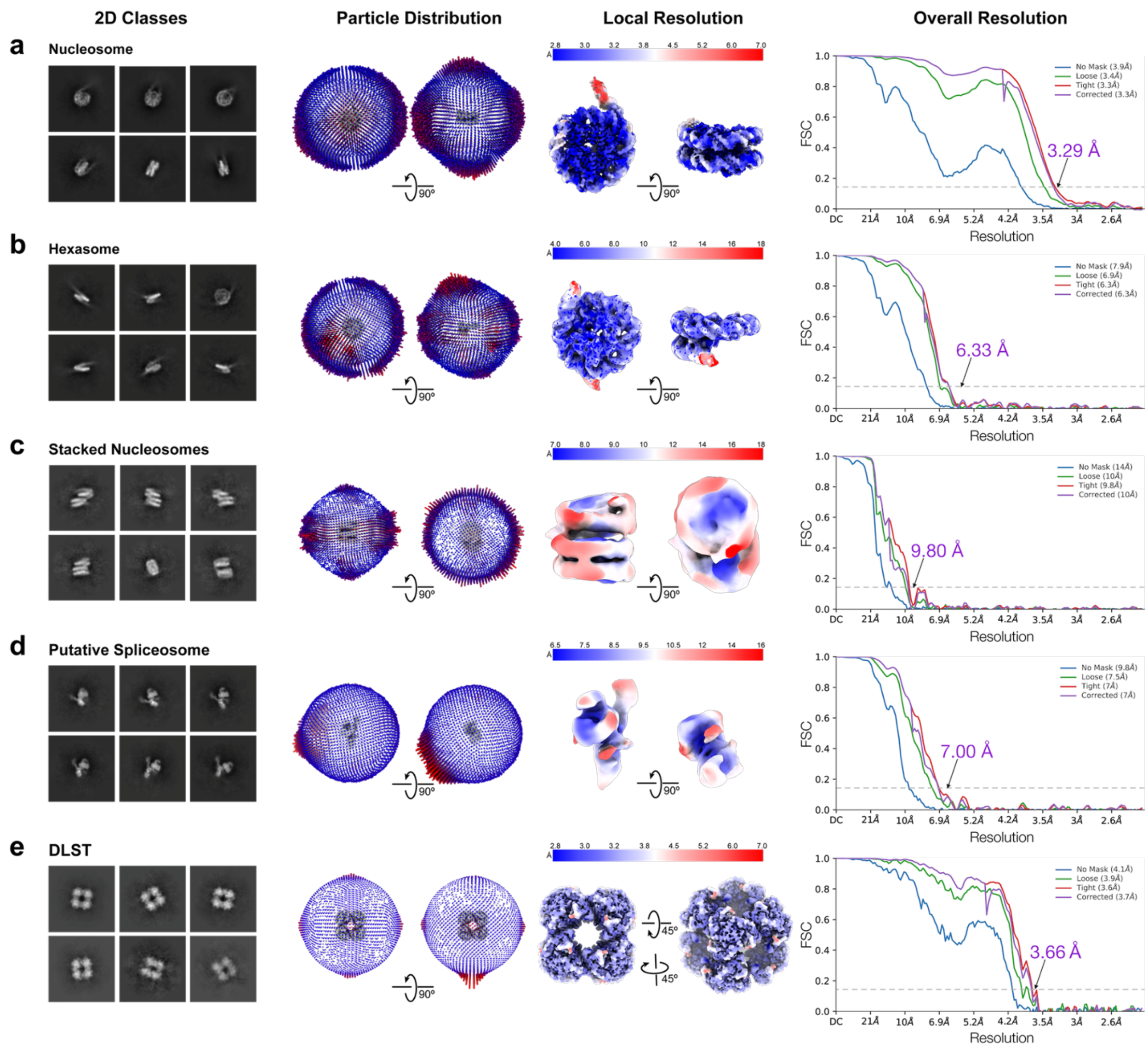

Supplementary Figure 2. Cryo-EM micrograph and analysis, continued.
(a-e) Statistics on final reconstructions of nucleosome (**a**), hexasome (**b**), di-nucleosomes (**c**), putative spliceosome (**d**) and DLST (**e**) including selected 2D class averages (left), Euler angle distribution of particles used in final reconstructions (second to left), local resolution maps with color ranging from blue to red representing high to low resolutions (second from right), and FSC curves calculated in CryoSPARC reported at the 0.143 FSC cutoff (right). In the Euler angle plot, the length of the cylinders is correlated to the distribution of particles in that direction. In the local resolution visualization, the maps are colored locally according to the scale above.

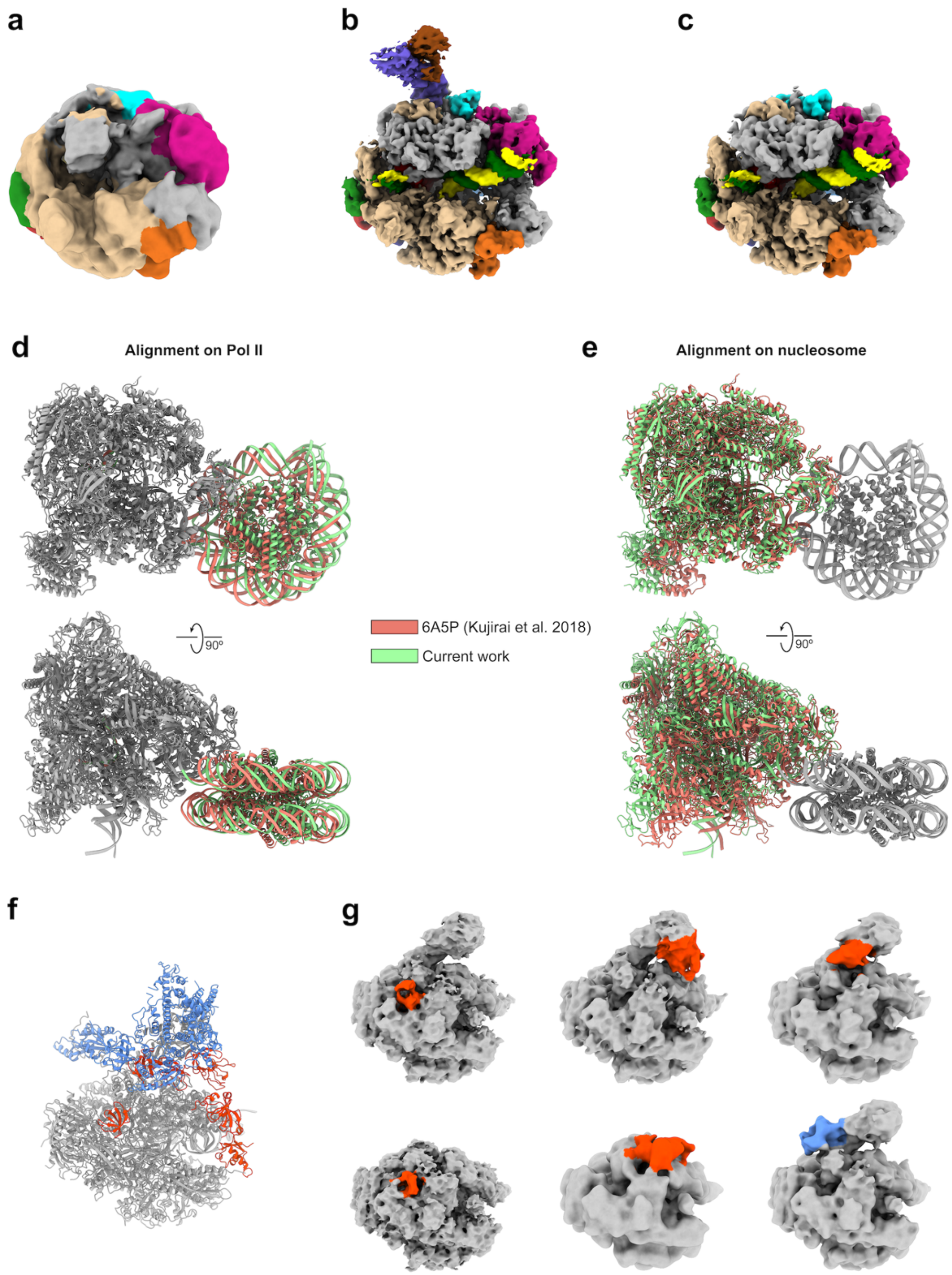

Supplementary Figure 3. Variability of cryo-EM densities of native EC.
(a-c) Cryo-EM densities of the apo-form Pol II (**a**), EC with the stalk **(b)** and EC without the stalk **(c)** with each subunit colored as described in **Figure 3. (d-e)** Comparison of nucleosome ECs from *Drosophila* (green, this study) and yeast paused at SHL(–5) (red, PDB: 6A5P) [8] with structures aligned on Pol II **(d)** and the nucleosome **(e)**. **(f)** Ribbon model of mammalian Pol II with elongation factor DSIF in red and histone chaperone SPT6 in blue (PBD: 6GMH [16]). **(g)** 3D classification of the Drosophila Pol II EC data focused on the DSIF or Spt6 binding sites reveal low resolution densities showing that some binding sites are occupied (red, DSIF; blue, Spt6), which may indicate co-purification of DSIF and Spt6 with endogenous Pol II. Mass spectrometry detected DSIF and Spt6 in high abundance.

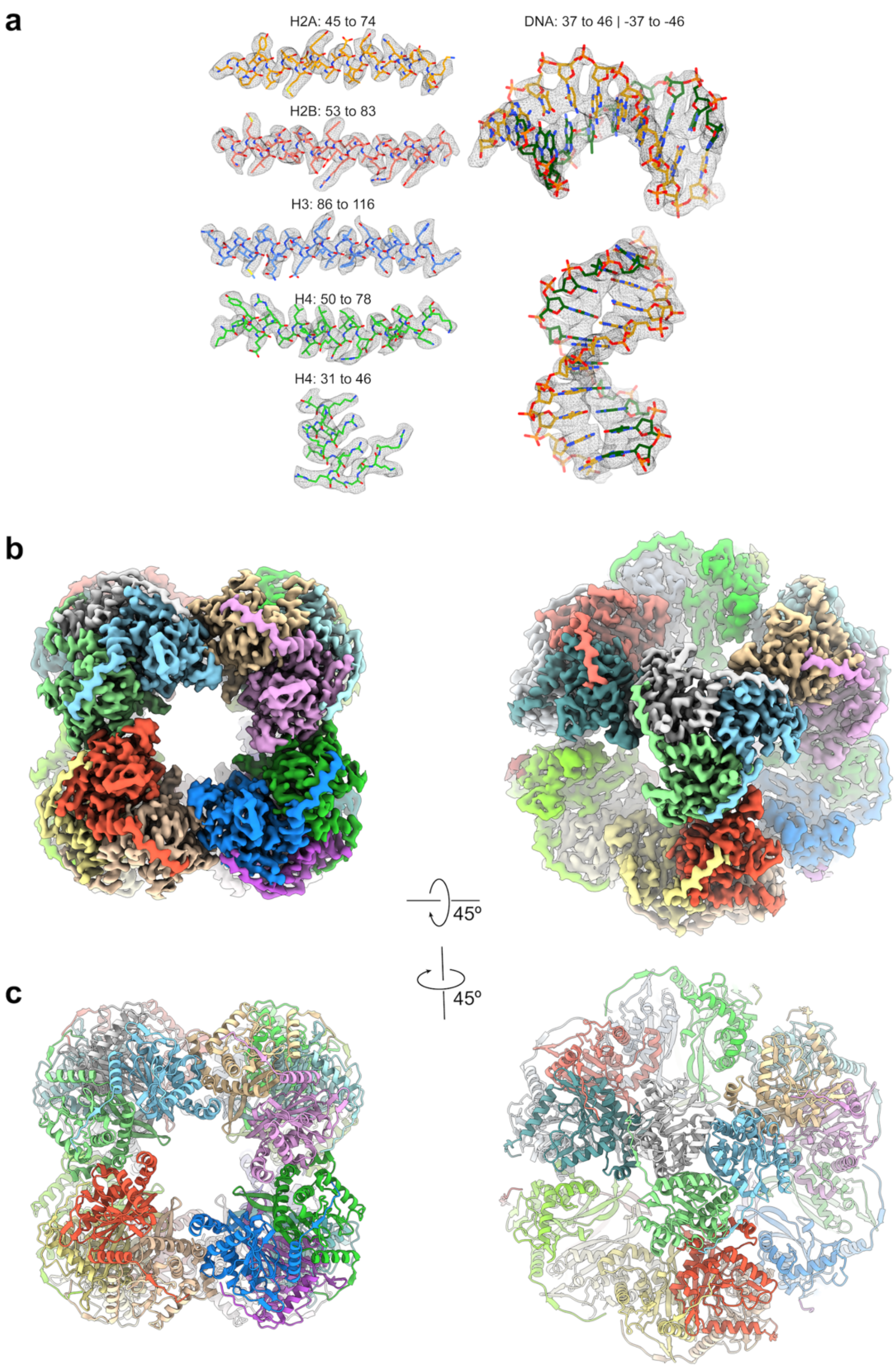

Supplementary Figure 4. Quality of cryo-EM of nucleosome reconstruction; Cryo-EM map and model of DLST.
(a) Selected representative 3.29 Å resolution cryo-EM densities of core histones and DNA in nucleosome superimposed on the refined models. **(b)** Two views of the cryo-EM density of *Drosophila* dihydrolipoyllysine-residue succinyltransferase component of mitochondrial 2-oxoglutarate dehydrogenase complex at 3.66 Å resolution, with individual subunits colored separately. **(c)** Two views of the refined model of DLST with individual subunits colored separately.

Supplementary Figure 5. Uncropped and unprocessed MNase digestion ethidium-bromide-stained gel from Figure 1b.

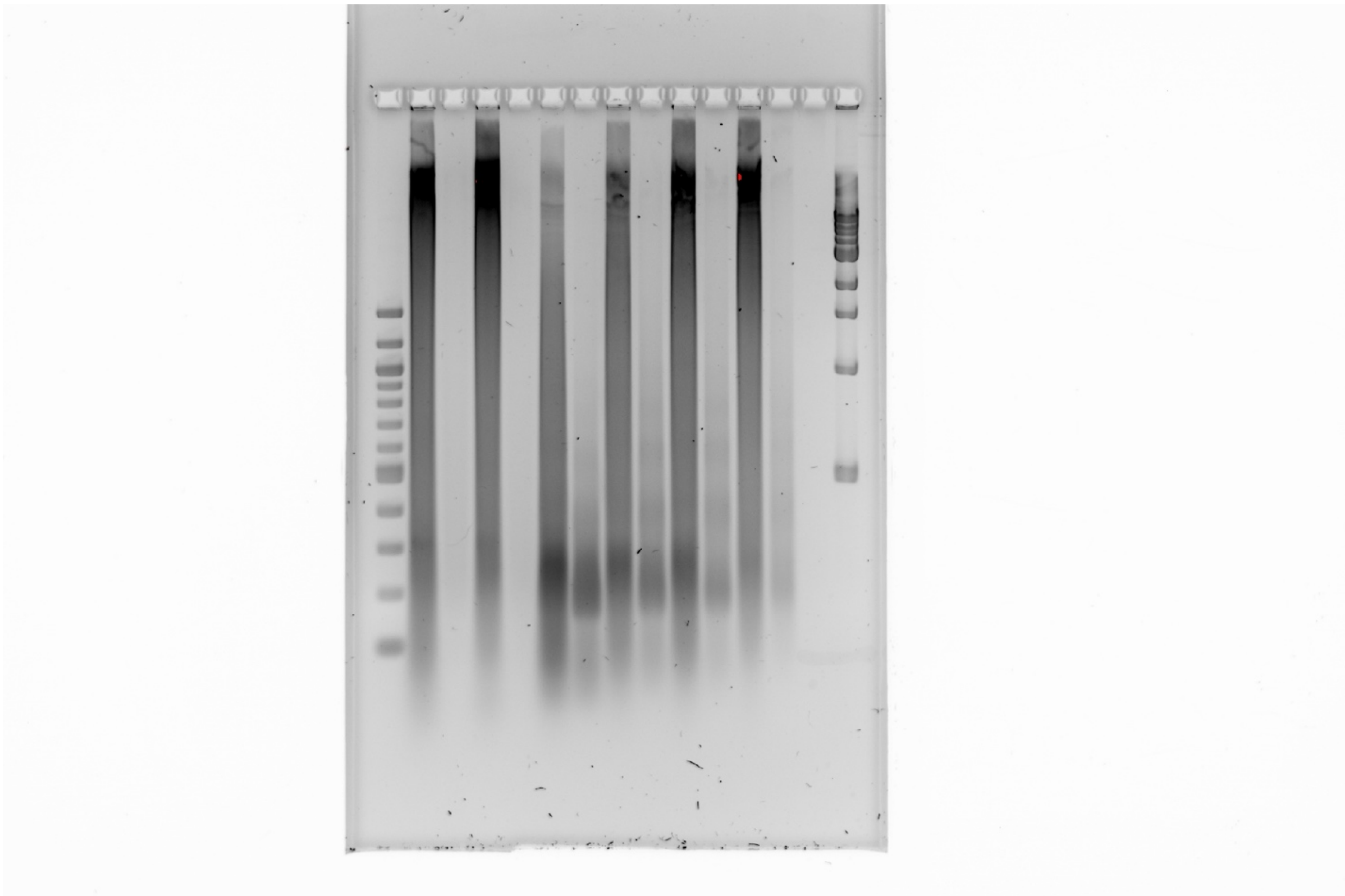

Supplementary Figure 6. Uncropped and unprocessed Coomassie-stained SDS-PAGE of affinity-purified samples from Figure 1c.

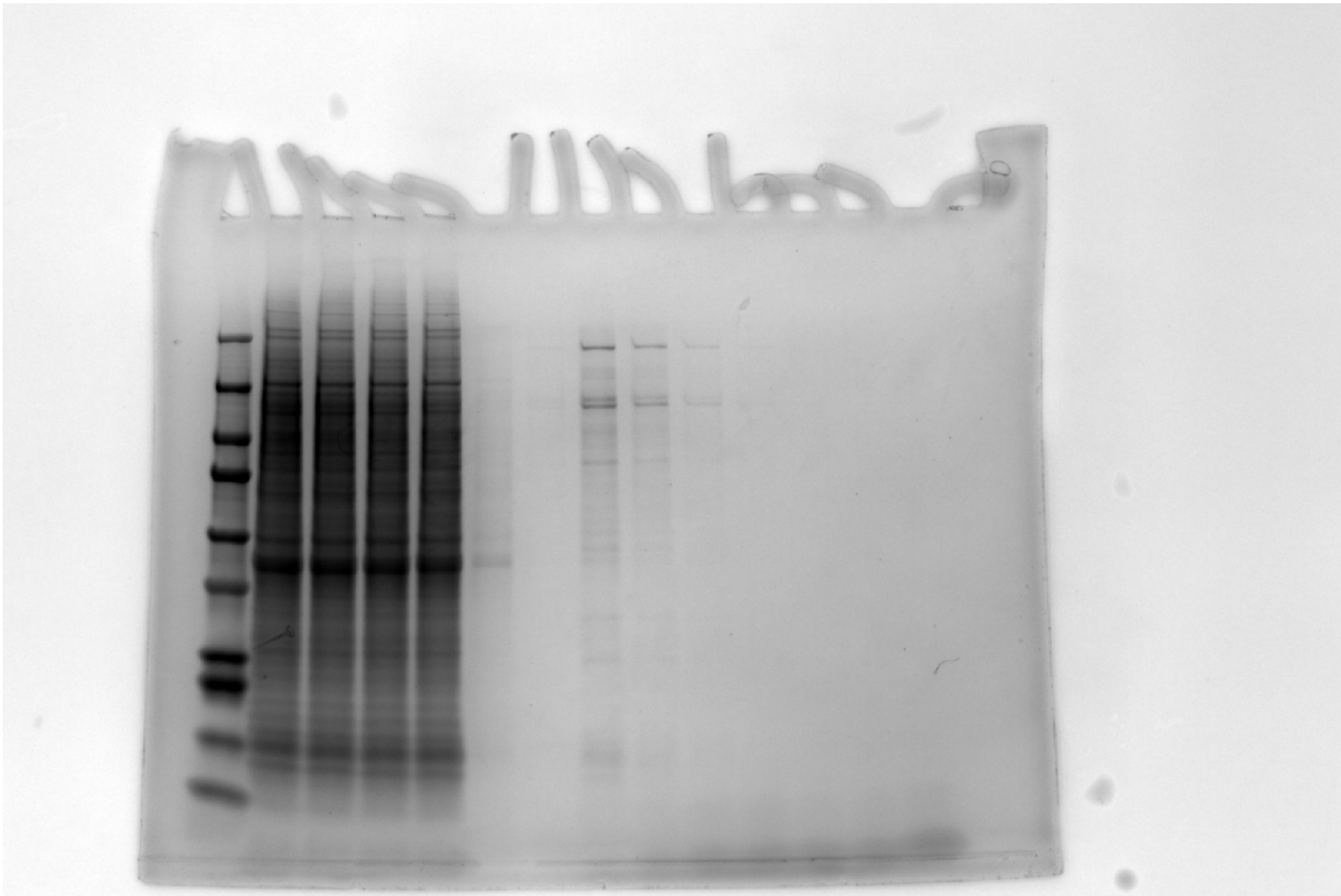

Supplementary Figure 7. Uncropped and unprocessed Western blot from Figure 1d.

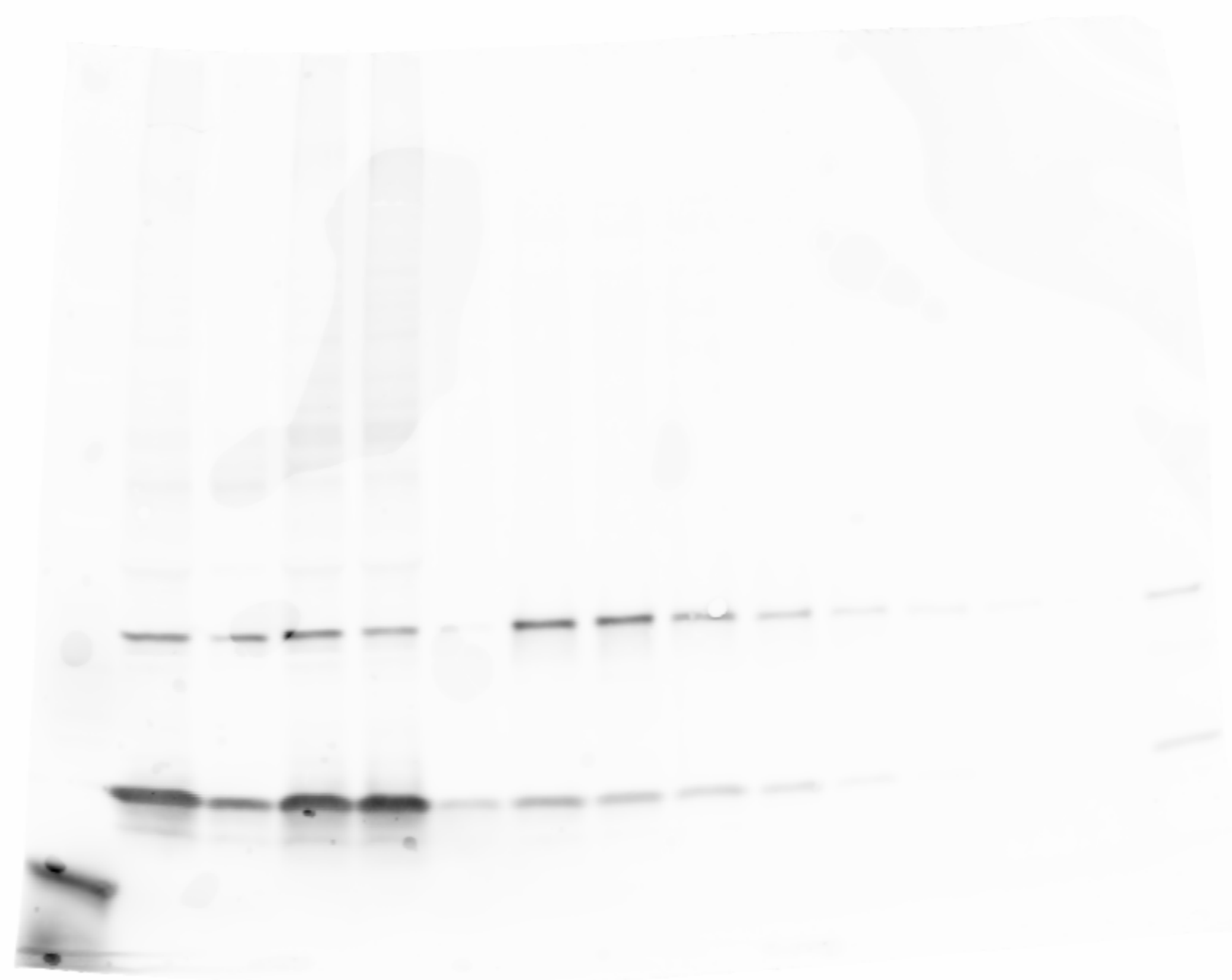

Supplementary Table S1. Proteins identified in the FLAG affinity-purified samples by liquid chromatography tandem mass spectrometry.

In gray, Pol II subunits below the selected Score Sequest HT threshold of 10.

	Uniprot ID	Gene Name	Description	Coverage [%]	# Peptides	# PSMs	MW [kDa]	Score Sequest HT	Phosphorylation (S; T; Y)	Methyl-ation (K)
RNA Polymerase II subunits	P04052	Polr2A	DNA-directed RNA polymerase II subunit RPB1	33	50	106	209	255.91	S1130; T1132	K902; K906; K1125; K1127
	A0A0B4 LI86	Polr2B	DNA-directed RNA polymerase II subunit RPB2	38	38	92	134	219.95		K131
	O97183	Polr2C	DNA-directed RNA polymerase II subunit RPB3	40	7	20	31.2	52.39		
	Q9VEA5	Polr2D	DNA-directed RNA polymerase II subunit RPB4	19	2	2	16.2	5.53		
	Q7JZF5	Polr2E	DNA-directed RNA polymerase II subunit RPB5	27	5	12	24.5	29.11	S113; S117	K124
	Q9VFB5	Polr2G	DNA-directed RNA polymerase II subunit RPB7	13	2	3	19.1	6.24		
	Q9VNZ3	Polr2H	DNA-directed RNA polymerases II subunit RPB8	35	4	12	17.2	20.66		
	P36958	Polr2I	DNA-directed RNA polymerase II subunit RPB9	60	6	15	15.1	37.6		
	Q9VJE4	Polr2J	DNA-directed RNA polymerase II subunit RPB11	9	1	1	13.4	2.13		
	Q9VC49	Polr2L	DNA-directed RNA polymerases II subunit RPB10	18	1	1	7.7	2.59		
Histon	Q4AB57	His2A	Histone H2A	60	5	19	13.4	39.76		
	A0A0B4 KH25	His2Av	Histone H2A variant	27	4	16	15	33.87		
	P84040	His4	Histone H4	57	8	28	11.4	48.81	Y89	K32
Transcription Factors	Q9VP05	MED1	Mediator of RNA polymerase II transcription subunit 1	6	6	6	149.4	16.15	T396; S399; S400; Y513; S667	
	Q9Y149	MED15	Mediator of RNA polymerase II transcription subunit 15	10	4	6	80.5	12.92	S521; T531; T538; T642; S644	
	Q9VEC1	MED17	Mediator of RNA polymerase II transcription subunit 17	17	5	7	71.5	14.12	S558; S559; S564; S567	
	Q05913	TfIIFalpha	General transcription factor IIF subunit 1	26	8	11	64.4	28	Y513	K196; K364; K365; K525
	Q9TVQ5	Spt4	Transcription elongation factor SPT4	30	2	4	13.3	11.95		
	Q9V460	Spt5	Transcription elongation factor SPT5	27	22	39	119.4	107.02		
	N0D8I3	Spt6	Transcription elongation factor spt6	22	26	37	208.5	95.28	S124; S335	K332
	Q05344	Ssrp	FACT complex subunit Ssrp1	35	20	54	81.5	117.2	S443; S628	K550; K554; K556
	Q8IRG6	dre4	FACT complex subunit spt16	26	24	57	123.5	118.78	S994	K423; K424; K910
	Q9VDY6	Polr2M	Gdown1	20	7	8	41.7	19.08		
Other	P15348	Top2	DNA topoisomerase 2	12	14	18	164.3	49.34		
	A0A0B4 K6K1	Rm62	RNA helicase	16	6	10	52.5	24.51		
	Q494M1	Top1	DNA topoisomerase I	9	7	9	111.8	20.38	T815; T821	K820
	P25171	Rcc1	Regulator of chromosome condensation	12	4	6	58.8	12.64	T286; T290	K289
	Q9V9T4	Acf	ATP-dependent chromatin assembly factor large subunit	4	4	4	170.3	11.24	Y519; Y640	
	Q9VW52	Prp3	Precursor RNA processing 3, isoform A	10	3	5	67.3	10.69	S23; S25; S62; S64; S65; T110	K18; K69; K92; K115
	Q9VEN2	cal1	Chromosome alignment defect 1	9	4	6	109.5	10.53	S488; S634; T637; T667; S681; T872; S888	K469; K470; K658; K672; K679; K898

	M9PC89	snRNP-U1-70K	U1 small nuclear ribonucleoprotein 70 kDa	8	3	6	52.9	19.69		
	P43332	snf	U1 small nuclear ribonucleoprotein A	15	2	4	24.5	11.07		

Supplementary Table S2. Cryo-EM data collection, interpretation and visualization details

Data Collection	
Microscope	Talos Arctica
Voltage (kV)	200
Camera	Falcon 4
Magnification	150,000
Electron exposure (e^-/Å^2)	50.65
Defocus range (μm)	-0.8 to -2.0
Pixel size (Å)	0.944
Number of Frames	42
Number of Images	31,103
Images/Hole	2
Data Collection Software	EPU v. 3.4
Data Processing	
Motion Correction	CryoSPARC v 4.3.1 *Patch Motion Correction (Multi)*
Data Processing	CryoSPARC v 4.3.1 – 4.5.1; Relion v 5.0
Data Interpretation	
Initial Models	AlphaFold 2; AlphaFold 3
Rigid Body Fit	UCSF ChimeraX v.1.8; Coot v 0.9.8
Model Building	Coot v 0.9.8
Model Refinement	Coot v 0.9.8 Real Space Refine; phenix.real_space_refine
Data Visualization	UCSF ChimeraX v.1.8
Figure Preparation	Apple Keynote; MS PowerPoint; Adobe Illustrator

Supplementary Table S3. Cryo-EM data collection, refinement and validation statistics for EC and Nucleosome EC.

	Pol II Overall (EMD-48624)	EC with stalk (EMD-48622) (PDB 9MU7)	EC without stalk EMD-48623 (PDB 9MU8)	Nucleosome EC ** (EMD-48625) (EMD-48626) (PDB 9MU9)
**Data collection and processing**				
Magnification	150,000	150,000	150,000	150,000
Voltage (kV)	200	200	200	200
Electron exposure (e–/Å^2)	50.65	50.65	50.65	50.65
Defocus range (μm)	0.8 to 2.0	0.8 to 2.0	0.8 to 2.0	0.8 to 2.0
Pixel size (Å)	0.944	0.944	0.944	0.944
Symmetry imposed	C1	C1	C1	C1
Initial particle images (no.)	876,764	876,764	876,764	36,552
Final particle images (no.)	68,716	70,116	96,409	36,552
Map resolution (Å)	2.86	3.44	3.37	7.79/4.31/6.12*
FSC threshold	0.143	0.143	0.143	0.143
Map resolution range (Å)	20-2.86	20-3.44	20-3.37	20-7.79/4.31/6.12
Map sharpening *B* factor (Å^2)	47.8	64.0	51.7	161.8/84.7/385.9
**Refinement**				
Initial model used (PDB code)	-	6GML	6GML	6GML/3LZ0
Model resolution (Å)	-	3.44	3.37	4.31/6.12
FSC threshold	-			
Model composition				
Non-hydrogen atoms	-	33,887	31,485	42,423
Protein residues	-	3,992	3,689	4,446
Nucleic acid residues	-	99	99	347
Ligands	-	Zn: 8	Zn: 8	Zn: 8
B factors (Å^2)	-			
Protein	-	13.6/491.5/117.2	57.2/403.6/112.2	30.0/1229.4/457.3
DNA	-	39.0/536.2/171.1	39.0/536.2/171.1	200.1/1104.7/352.9
Ligand	-	127.7/181.2/156.5	127.7/181.2/156.5	286.1/944.0/570.4
R.m.s. deviations				
Bond lengths (Å)	-	0.007	0.005	0.004
Bond angles (°)	-	1.094	1.077	1.016
Validation				
MolProbity score	-	1.48	1.48	1.48
Clashscore	-	4.50	4.54	5.05
Poor rotamers (%)	-	0.00	0.00	0.00
Ramachandran plot				
Favored (%)	-	96.29	96.24	96.69
Allowed (%)	-	3.71	3.71	3.29
Disallowed (%)	-	0.00	0.00	0.02

* left - overall resolution; middle - focused on Pol II; right - focused on nucleosome

** validation performed against the nucleosome EC composite map

Supplementary Table S4. Cryo-EM data collection, refinement and validation statistics for nucleosome, hexasome and DLST

	Nucleosome (EMD-48619) (PDB 9MU4)	Hexasome (EMD-48620) (PDB 9MU5)	DLST EMD-48621 (PDB 9MU6)
**Data collection and processing**			
Magnification	150,000	150,000	150,000
Voltage (kV)	200	200	200
Electron exposure (e–/Å^2)	50.65	50.65	50.65
Defocus range (μm)	0.8 to 2.0	0.8 to 2.0	0.8 to 2.0
Pixel size (Å)	0.944	0.944	0.944
Symmetry imposed	C1	C1	
Initial particle images (no.)	1,848,096	231,425	70,106
Final particle images (no.)	66,988	38,738	23,543
Map resolution (Å)	3.29	6.33	3.66
FSC threshold	0.143	0.143	0.143
Map resolution range (Å)	20-3.29	20-6.33	20-3.66
Map sharpening *B* factor (Å^2)	50.7	471.2	102.6
**Refinement**			
Initial model used (PDB code)	3LZ0	Octameric Nucleosome	AlphaFold
Model resolution (Å)	3.29	6.33	3.66
FSC threshold	0.143	0.143	0.143
Model composition			
Non-hydrogen atoms	12,794	9,666	43,134
Protein residues	770	532	5,616
Nucleic acid residues	328	266	-
Ligands			
B factors (Å^2)			
Protein	23.6/168.3/67.5	124.0/1398.6/616.9	40.6/144.90/78.1
DNA	15.7/472.4/123.3	188.7/1043.1/444.0	-
Ligand	-	-	-
R.m.s. deviations			
Bond lengths (Å)	0.006	0.005	0.006
Bond angles (°)	0.971	0.844	1.239
Validation			
MolProbity score	1.28	1.38	1.86
Clashscore	2.50	4.86	7.51
Poor rotamers (%)	0.00	0.00	0.00
Ramachandran plot			
Favored (%)	96.29	97.31	93.09
Allowed (%)	3.71	2.69	6.91
Disallowed (%)	0.00	0.00	0.00

Supplementary Table S5. Protein sequences used for model building

Protein Name	Gene Name	Uniprot ID
DNA-directed RNA polymerase II subunit RPB1	RPB1	P04052
DNA-directed RNA polymerase II subunit RPB2	RPB2	P08266
DNA-directed RNA polymerase II subunit RPB3	RPB3	O97183
DNA-directed RNA polymerase II subunit RPB4	RPB4	Q9VEA5
DNA-directed RNA polymerase II subunit RPB5	RPB5	Q7JZF5
DNA-directed RNA polymerase II subunit RPB6	RPB6	Q24320
DNA-directed RNA polymerase II subunit RPB7	RPB7	Q9VFB5
DNA-directed RNA polymerase II subunit RPB8	RPB8	Q9VNZ3
DNA-directed RNA polymerase II subunit RPB9	RPB9	P36958
DNA-directed RNA polymerase II subunit RPB10	RPB10	Q9VC49
DNA-directed RNA polymerase II subunit RPB11	RPB11	Q9VJE4
DNA-directed RNA polymerase II subunit RPB12	RPB12	Q6IGE3
Histone H2A	H2A	P84051
Histone H2B	H2B	P02283
Histone H3	H3	P02299
Histone H4	H4	P84040
Dihydrolipoyllysine-residue succinyltransferase component of 2-oxoglutarate dehydrogenase complex, mitochondrial	DLST	Q9VGQ1
