# Supplementary material for: Structural Characterization of Native RNA Polymerase II Transcription Complexes and Nucleosomes in Drosophila melanogaster
Source: Nat Commun. 2026 Aug 5;17:9616. doi: 10.1038/s41467-026-75963-0 (PMC13554098; doi:10.1038/s41467-026-75963-0)
Supplement: Supplementary file 2 [file 41467_2026_75963_MOESM2_ESM.pdf]

Description of Additional Supplementary Files

Supplementary Movie 1. Cryo-EM density map of EC with and without Rpb4/7 stalk.

Overview of the cryo-EM maps of the EC with and without the stalk. Related to Fig. 3A

Supplementary Movie 2. Cryo-EM density map of nucleosome EC.

Overview of the cryo-EM composite map of the nucleosome EC. Related to Fig. 5
